# Case Report: Paracentral acute middle maculopathy following carotid artery dissection

**DOI:** 10.3389/fcvm.2025.1560482

**Published:** 2025-06-06

**Authors:** Muhi Dean Barazi, Yusuf Bade, Malek Zanbrakji, Patrick A. Stone, Omar Belal Sabbagh, Mohsin H. Ali, Alexander Melamud

**Affiliations:** ^1^ Biosciences Department, Rice University, Houston, TX, United States; ^2^ School of Medicine, George Washington University, Washington, DC, United States; ^3^ Biology Department, Georgetown University, Washington, DC, United States; ^4^ Department of Vascular Surgery, Vanderbilt University, Nashville, TN, United States; ^5^ Biology Department, West Virginia University, Morgantown, WV, United States; ^6^ Private Practitioner, Reston, VA, United States

**Keywords:** paracentral acute middle maculopathy, carotid, dissection, optical coherence tomography, magnetic resonance imaging, retinal artery, artery occlusion, case report

## Abstract

**Background:**

Paracentral acute middle maculopathy (PAMM) is a rare, presumed ischemic maculopathy. While primarily associated with retinal vascular pathologies, several case studies have documented PAMM diagnoses following systemic cardiovascular events or interventions. Here, we discuss a case of PAMM development after carotid artery dissection (CAD).

**Case presentation:**

A woman in her late 30 s presented to the emergency department with transient right-side weakness and amaurosis in her left eye lasting 1–2 h. An initial stroke and embolic workup showed no significant findings. She later presented to the retina clinic with normal visual acuity and intraocular pressure. Dilated fundus examination, intravenous fluorescein angiography, and spectral-domain optical coherence tomography (SD-OCT) were unremarkable. Several days later, the patient returned to the emergency department complaining of transient ride-sided paresthesia, transient facial weakness, and dysarthria. A computed tomography angiogram revealed a dissection of the left internal carotid artery. Repeated retinal evaluation revealed a hyperreflective band on SD-OCT, characteristic of PAMM, spanning from the inner plexiform layer to the outer plexiform layer. Subsequent SD-OCT scans showed a resolution of the acute hyperreflective PAMM lesion with corresponding attenuation of the affected inner nuclear layer. Follow-up visits indicated a residual inferior paracentral scotoma in the affected eye.

**Conclusions:**

This case illustrates the rare occurrence of PAMM associated with CAD, underscoring the link between systemic vascular events and retinal ischemia, and demonstrating the potential of PAMM as an early indicator of the causative vascular pathology.

## Introduction

Paracentral acute middle maculopathy (PAMM) encompasses a range of presumed ischemic maculopathies, characterized by hyperreflective, band-like lesions in the middle retinal layers, which lead to subsequent atrophy (1). Although the exact etiology of PAMM is not entirely understood, these lesions are largely attributed to transient reductions in blood flow, resulting in ischemia in the intermediate (ICP) and deep capillary plexus (DCP) layers of the middle retina (1, 2).

PAMM has been associated with various retinal vascular pathologies (3–6), and, interestingly, with mixed evidence, systemic vascular risk factors or conditions (7–9). Furthermore, a recent retrospective study indicated that patients with isolated PAMM have a higher likelihood of receiving a cardiovascular disease diagnosis (10). Several case reports have documented the occurrence of PAMM following events such as coronary angiography, internal carotid endovascular repair, or ischemic cardiomyopathy (11–14). To our knowledge, this case report presents the first documented case of PAMM associated with carotid artery dissection (CAD).

## Case presentation

A woman in her late 30s with a history of congenital aqueductal stenosis and intrauterine growth restriction presented to the emergency department at a Level One Trauma Center in mid-December 2023. Her complaints included right-side weakness and three distinct episodes of transient amaurosis in her left eye, each lasting approximately 20 min over the course of two hours. She also relayed transient paresthesia in her right arm and leg. Her family history included glaucoma (grandmother) and diabetes, uterine/breast cancer, and heart disease (mother). The patient was right-handed, a non-smoker, and did not consume alcohol. She denied any history of hypertension, trauma, head and neck surgery, fibromuscular dysplasia, or chiropractic manipulation. She did report a recent history of influenza accompanied by a severe cough, with her symptoms emerging immediately after a coughing spell. A full timeline of the patient's history with events is provided in Table 1.

**Table 1:** Full timeline of the patient's history.

Date	Event
December 15	The patient presented to the emergency department with right-side weakness, episodes of transient amauorosis (left eye), and transient right arm and leg paresthesia lasting approximately two hours The patient was admitted to the hospital
December 16	CT, MRI, and magnetic resonance angiogram of the neck with and without contrast were performed, and no abnormal relevant findings were observed The patient was discharged for follow-up with ophthalmology
December 21	The patient presented to the outpatient retina clinic reporting amaurosis lasting for 24 h before the visit DFE, SD-OCT, and IVFA were performed without abnormal results The patient was discharged for recommended follow-up with neurology
December 26	The patient presented again to the emergency department with worsening right-sided paresthesia, facial droop, and dysarthria First CTA was performed, and the patient was diagnosed with dissection of the left internal carotid artery The patient was discharged and prescribed clopidogrel, atorvastatin, low-dose aspirin, and magnesium Follow-up imaging was recommended
December 29	The patient returned to the emergency department to reassess persistent symptoms CTA, CT, and MRI were non-progressive The patient was discharged for outpatient follow-up
January 4	The patient returned to the retina clinic DFE, SD-OCT, and IVFA were performed SD-OCT revealed a hyperreflective band in the inner retina consistent with PAMM
February 5	The patient returned to the retina clinic for a follow-up visit DFE and SD-OCT were performed SD-OCT showed improvements from PAMM
February 26	The patient returned to the retina clinic Humphrey visual field testing revealed a scotoma on the left eye
April 1	The patient returned to the retina clinic for a follow-up visit DFE and SD-OCT were performed SD-OCT showed no worsening from the previous visit
August 12	The patient returned to the retina clinic for a follow-up visit DFE, SD-OCT, and IVFA were performed PAMM lesion was similar to the previous visit, and inner nuclear layer attenuation was observed along residual scotoma

CT, computed tomography; CTA, computed tomography angiogram; DFE, dilated fundus examination; IVFA, intravenous fluorescein angiography; MRI, magnetic resonance imaging; PAMM, paracentral acute middle maculopathy; SD-OCT, spectral-domain optical coherence tomography.

The patient was admitted to undergo a stroke and embolic workup. Computed tomography (CT) without contrast of the head revealed enlargement of the lateral and third ventricles, suggestive of hydrocephalus; no acute territorial infarction, hemorrhage, or obstructing lesions were detected. Magnetic resonance imaging (MRI) of the brain and orbits with and without contrast indicated diffuse ventriculomegaly suggestive of communicating hydrocephalus but showed no evidence of obstructing mass lesions or abnormal subependymal or leptomeningeal enhancement. There were no signs of acute decompensated hydrocephalus, intracranial masses, acute hemorrhages or infarctions, or orbital abnormalities. Additionally, a magnetic resonance angiogram (MRA) of the head and neck (without contrast), as well as an MRA of the neck (without and with contrast) revealed no stenosis of the proximal right or left internal carotid arteries, no intracranial large vessel occlusion, and no hemodynamically significant arterial stenosis; the vertebrobasilar arterial system was patent (Supplementary Figure S1). However, the MRI protocol did not include diffusion-weighted imaging or fat-suppressed T1-weighted sequences, which may have limited the sensitivity for detecting a mural hematoma or other early features of dissection. A chest x-ray was normal. Laboratory tests, including a complete blood count, metabolic panel, liver function tests, and pregnancy testing, were all negative. She tested positive for Influenza A but negative for Influenza B and SARS-CoV-2. The patient was evaluated by the Neurology and Neurosurgery teams, who recommended outpatient follow-up without immediate intervention based on the imaging results. The consulting Neurologist did not diagnose transient ischemic events nor advise any anti-thrombotic therapy at that time. She was discharged with advice to follow up with an ophthalmologist.

**Figure 1:**
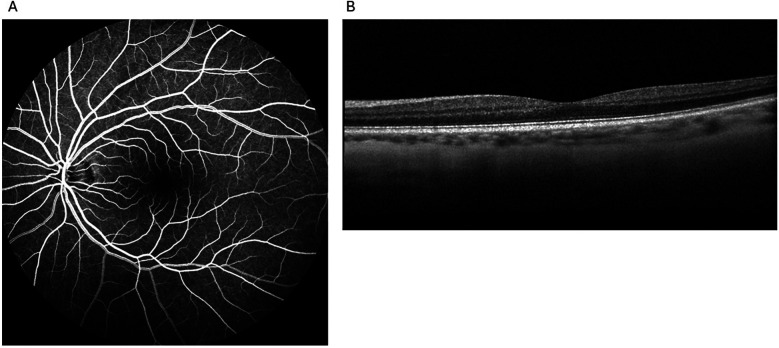
Unremarkable examination results without any evidence of paracentral acute middle maculopathy in the affected eye from the first visit to the retina clinic: intravenous fluorescein angiography **(A)** and spectral-domain optical coherence tomography **(B)**.

She presented to the outpatient retina clinic five days later reporting blurred vision and ‘brown stuff’ floating intermittently in her left eye for about a week and vision obscurations 24 h before the visit. Her visual acuity was 20/20, and bilateral intraocular pressure was normal. Dilated fundus examination (DFE), intravenous fluorescein angiography (IVFA), and spectral-domain optical coherence tomography (SD-OCT) were performed. No optic disc edema or pallor, retinal nerve fiber layer defects, hemorrhages, holes or tears, macular edema, or subretinal fluid were observed on ophthalmoscopy. IVFA demonstrated normal arteriovenous (AV) transit time. SD-OCT results were also within normal limits (Figure 1). She was advised to follow up with her neurologist.

**Figure 2:**
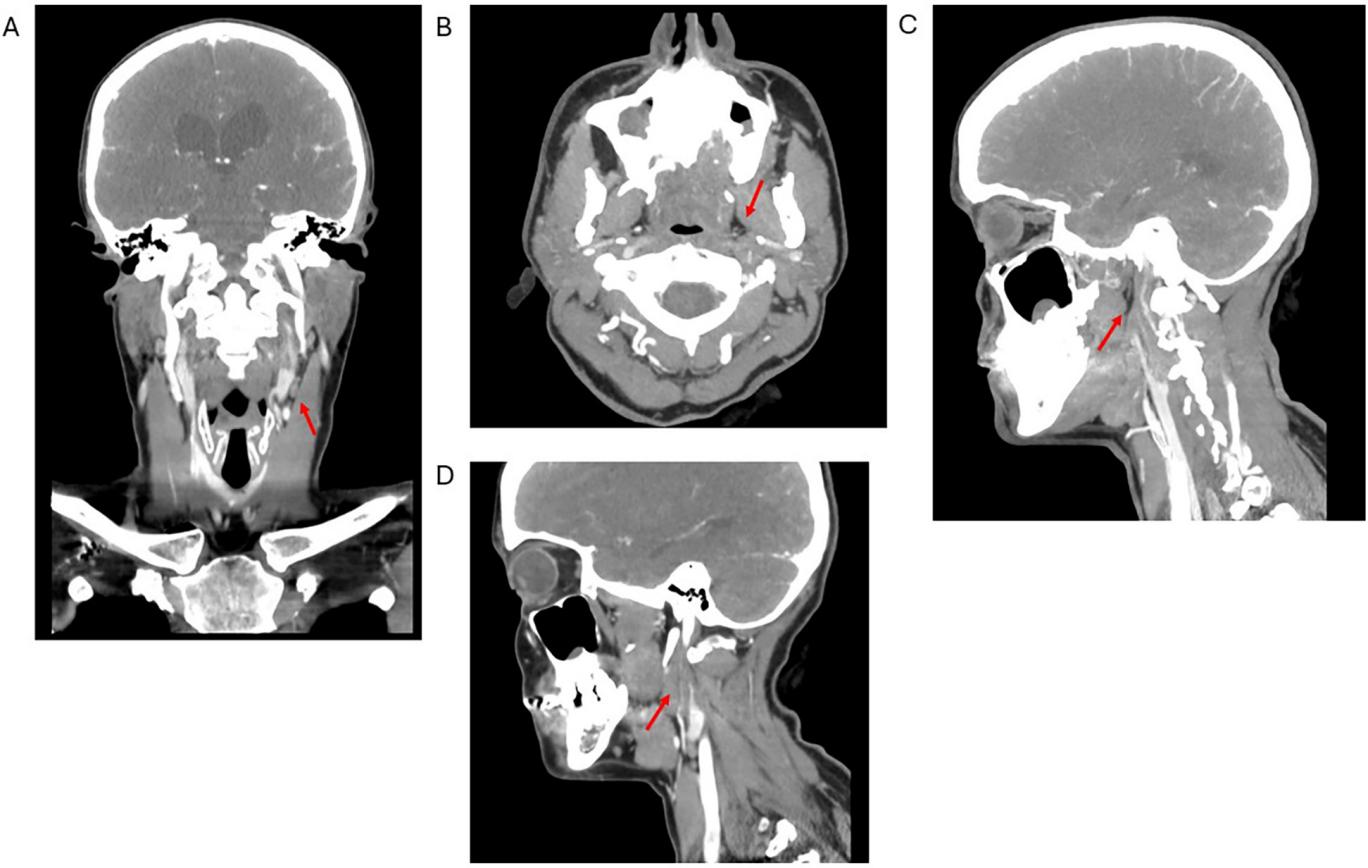
Computed tomography angiogram of the head and neck in coronal **(A)**, axial **(B)**, sagittal distal **(C)**, and sagittal proximal **(D)** views. Red arrows show dissection of the left internal carotid artery with severe stenosis of the true lumen and false lumen thrombus extending through the mid and distal portions of the vessel.

Five more days later, the patient developed worsening right-sided paresthesia, transient facial droop, and dysarthria, prompting her return to the emergency department. A computed tomography angiogram (CTA) of the head and neck with contrast revealed interval development of extensive severe stenosis in the post-bulbar segment of the left internal carotid artery (ICA), with marked luminal narrowing and irregularity extending through the mid-to-distal vessel segments, consistent with a left ICA dissection (Figure 2). The terminal cervical segment and intracranial portion of the left ICA, as well as the right common carotid and ICAs, remained patent. A repeat MRI of the brain was performed to rule out intracranial infarction and was negative. The patient was monitored for clinical stability and discharged three days later with prescriptions for daily oral clopidogrel (75 mg), atorvastatin (40 mg), low-dose aspirin (81 mg), and magnesium (250 mg), to be continued for at least six months. The etiology of her CAD was thought to possibly be related to her history of recent influenza and vigorous coughing as heavy coughing increases intra-abdominal and intrathoracic pressure (15). Upon discharge, the neurology team noted that the etiology of her right hemiparesis remained “unclear”, acknowledging that “small posterior brainstem infarcts could be missed” on imaging, and recommended repeat imaging during outpatient follow-up. Three days after this second hospital admission, the patient returned to the emergency department due to persistent symptoms. Repeat CTA of the head and neck, CT of the head without contrast, and MRI of the brain without contrast were non-progressive. She was discharged once again for outpatient follow-up.

**Figure 3:**
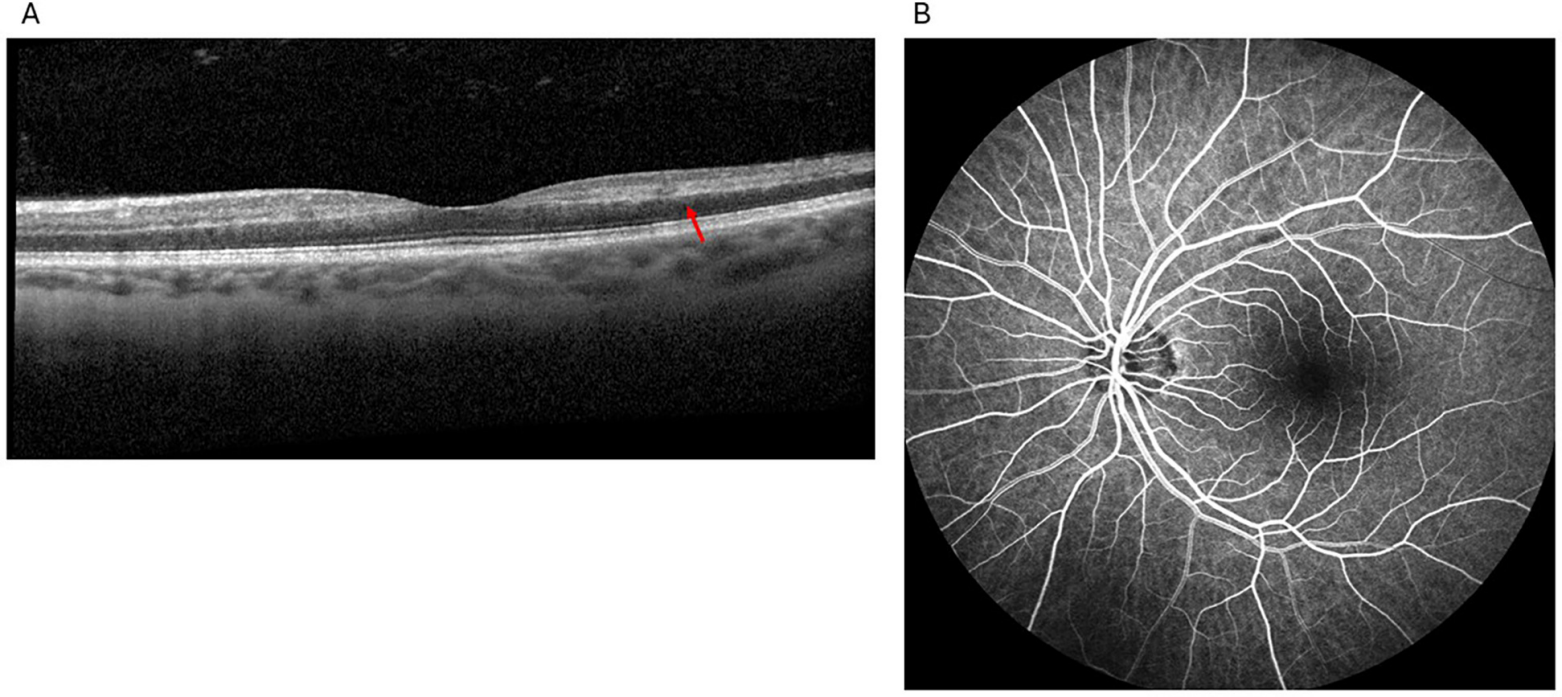
Findings from the second visit to the retina clinic: spectral-domain optical coherence tomography showed hyperreflectivity primarily involving the middle retinal layers, specifically the inner nuclear layer, extending into the nasal and temporal parafovea (red arrow), consistent with paracentral acute middle maculopathy **(A)**, fluorescein angiography showed normal arteriovenous transit time, no delayed arteriolar or venous filling, no areas of nonperfusion, and no leakage **(B)**.

The patient subsequently re-presented to the outpatient retinal clinic for further evaluation in early January noting a new scotoma. SD-OCT revealed a hyperreflective band extending from the inner plexiform layer (IPL) to the outer plexiform layer (OPL) (Figure 3) consistent with a PAMM diagnosis. DFE and IVFA findings were noncontributory with normal AV transit time, no delayed arteriolar or venous filling, no areas of nonperfusion, and no leakage (Figure 3). Brimonidine tartrate ophthalmic solution 0.15% was prescribed for thrice-daily use for a month. A follow-up with a neurologist was again recommended. The patient was also referred for visual field testing.

One month later, the patient reported sudden visual blurring shortly after her last appointment. SD-OCT showed a continued reduction of the PAMM lesion. The patient was instructed to discontinue brimonidine tartrate ophthalmic solution 0.15%. In late February, a Humphrey visual field (HVF) test revealed an inferior paracentral scotoma in the left eye, aligning with the patient's subjective vision loss. Two follow-up appointments in April and August demonstrated ongoing resolution of the PAMM lesion and progressive inner retinal atrophy (Supplementary Figure S2). The patient continued to complain of a persistent scotoma. A one-year follow-up was scheduled in the retina clinic.

## Discussion

This case presents a rare instance of PAMM in a woman in her late 30s likely associated with spontaneous CAD. The patient's initial symptoms included right-sided weakness, three episodes of amaurosis, each approximately 20 min in her left eye over the course of two hours, and transient paresthesia in her right arm and leg. The subsequent workup revealed significant findings underlining the complex interplay between systemic vascular events and retinal ischemia. PAMM is characterized by band-like hyperreflective parafoveal lesions at the level of the inner nuclear layer (INL), often extending from the IPL to the OPL (1, 5, 16, 17). Once considered a variation of acute macular neuroretinopathy, PAMM is now recognized as a distinct entity primarily linked to ischemia of the DCP and, to a lesser extent, the ICP (5, 16). The parafoveal region, a watershed zone with high oxygen demand, is particularly vulnerable to hypoperfusion (5). Over time, ischemic damage leads to chronic thinning and atrophy of the INL (1), as observed in this patient.

The patient's presentation included sudden onset scotomas without significant loss of visual acuity, a typical feature of PAMM (1, 2). While scotomas are often permanent, mild to moderate visual acuity reduction may also occur (1, 2, 5). DFE in PAMM can appear normal or reveal subtle parafoveal gray-white discoloration (1), although no ophthalmoscopic abnormalities were noted in this case. SD-OCT is crucial for confirming the diagnosis, revealing characteristic hyperreflective lesions in the acute phase and subsequent retinal thinning (18). En-face OCT can demonstrate patterns such as perivenular fern-like lesions, associated with milder disease, and globular patterns, linked to more severe ischemia (17, 19). IVFA is usually normal in PAMM, as it does not image the DCP (5), but it may be helpful to identify underlying conditions such as delayed arterial flow in partial central retinal artery occlusion (CRAO) (16, 17). In our case, IVFA was within normal limits.

PAMM is associated with various conditions causing insufficient blood flow within the DCP, including CRAO, central retinal vein occlusion (CRVO), and branch retinal artery occlusion (2, 18). Approximately 5% of eyes with CRVO exhibit evidence of PAMM (3). PAMM can also be the sole finding in incomplete CRAO or CRVO (2, 17, 18), underscoring the importance of a comprehensive evaluation for retinal vascular occlusions. Beyond retinal vascular events, PAMM has been linked to systemic factors such as hypercoagulable states, diabetes, hypertension, dyslipidemia, and autoimmune diseases (2, 5, 16, 18). It has also been associated with migraine, pregnancy, flu-like illnesses, and vasoconstrictive drugs like sumatriptan and synephrine (5, 16). Notably, PAMM may be the first sign of serious systemic conditions such as cardiovascular disease or giant cell arteritis, necessitating urgent evaluation (18).

In surgical contexts, PAMM has been reported following intraocular and extraocular procedures, including intravitreal injections, pars plana vitrectomy, and vascular surgeries like aortic aneurysm repair and cardiopulmonary bypass (2, 16). Vasoconstriction from periocular anesthesia or systemic vascular compromise likely contributes to these cases (16). This case is particularly significant as it establishes an association between PAMM and CAD, a connection not previously documented. CAD can disrupt blood flow in the ICA, affecting the ophthalmic artery, which supplies the retinal and choroidal vasculature. Microembolization or hypoperfusion stemming from CAD may compromise the OPL and INL, located in the retinal watershed zone and therefore particularly susceptible to ischemia (3, 20). Furthermore, reperfusion injury could also play a role in the retinal changes, contributing to damage in ischemic retinal tissue during the restoration of blood flow (16).

To our knowledge there has been only one other published case of PAMM associated with CAD (23). However, in this case published by Guleser, the PAMM was preceded by internal carotid stenting for CAD. Our case highlights PAMM in association with spontaneous CAD, likely secondary to severe coughing, and serves to highlight to the Ophthalmology community the need to consider CAD on the differential diagnosis of possible associations with PAMM. PAMM has also been previously documented in the context of internal carotid endovascular repair and coronary angiography, sometimes as the only presenting sign of ischemic cardiomyopathy or other vascular obstructions (11–14). PAMM has been associated with the ischemic cascade caused by progressive vascular obstruction (21). Given that PAMM can indicate serious underlying conditions, an immediate and thorough systemic workup should be considered (18). In the presence of PAMM without evidence of CRVO, CRAO, or other clear etiology, a workup for cardiovascular disease, carotid imaging, and assessment for inflammatory, infectious, or hypercoagulable states is prudent (2, 17, 18).

The treatment approach for this patient focused on addressing the primary systemic vascular issue, as specific treatments for PAMM are currently unavailable. Brimonidine tartrate ophthalmic solution 0.15% was empirically prescribed due to its potential neuroprotective effects (22), though its efficacy is uncertain. The patient was also prescribed antiplatelet medications and statins to reduce the risk of further embolic events, with regular ophthalmic follow-ups essential for monitoring her condition. Nonetheless, timely recognition and management of the dissection were pivotal in preventing more severe ischemic complications. It is worth mentioning that a CTA was not performed during the patient's initial visit to the emergency department, as the MRI findings were unremarkable. While the subsequent CTA during the second visit revealed the CAD, it remains uncertain whether an earlier imaging study might have identified the dissection at an earlier stage.

Finally, this case highlights the interplay between systemic vascular conditions and retinal ischemia, suggesting that PAMM may indicate an underlying vascular pathology. Clinicians should maintain a high index of suspicion in patients presenting with visual symptoms, as early identification and intervention in systemic vascular disorders can help prevent further ocular complications and improve overall patient outcomes. More research is warranted to explore the etiology of PAMM, its association with vascular conditions and risk factors, and potential therapeutic approaches.

This case report has important strengths. It contributes significantly by documenting a novel association between PAMM and spontaneous CAD, highlighting the importance of systemic vascular evaluations in patients presenting with PAMM. It benefits from detailed diagnostic imaging, longitudinal follow-up, and a comprehensive timeline of events, collectively providing a robust clinical narrative. However, the report is inherently limited by its single-patient scope, which restricts generalizability. Additionally, while the case provides insight into the systemic associations of PAMM, it does not delve deeply into potential therapeutic strategies or long-term outcomes, both of which warrant further research.

## Conclusion

This is the first case of a patient with PAMM following CAD without concurrent procedural intervention, and the second case we are aware of in a patient with CAD. This case not only contributes to the understanding of the relationship between PAMM and systemic vascular events, but it also underscores the potential need for comprehensive vascular evaluation and long-term ophthalmic follow-up in patients presenting with PAMM.

## Data Availability

The original contributions presented in the study are included in the article/Supplementary Material, further inquiries can be directed to the corresponding author.
