# Supplementary material for: Case Report: Paracentral acute middle maculopathy following carotid artery dissection
Source: Front Cardiovasc Med. 2025 Jun 6;12:1560482. doi: 10.3389/fcvm.2025.1560482 (PMC12179186; doi:10.3389/fcvm.2025.1560482)
Supplement: Supplementary file 1 [file Datasheet1.pdf]

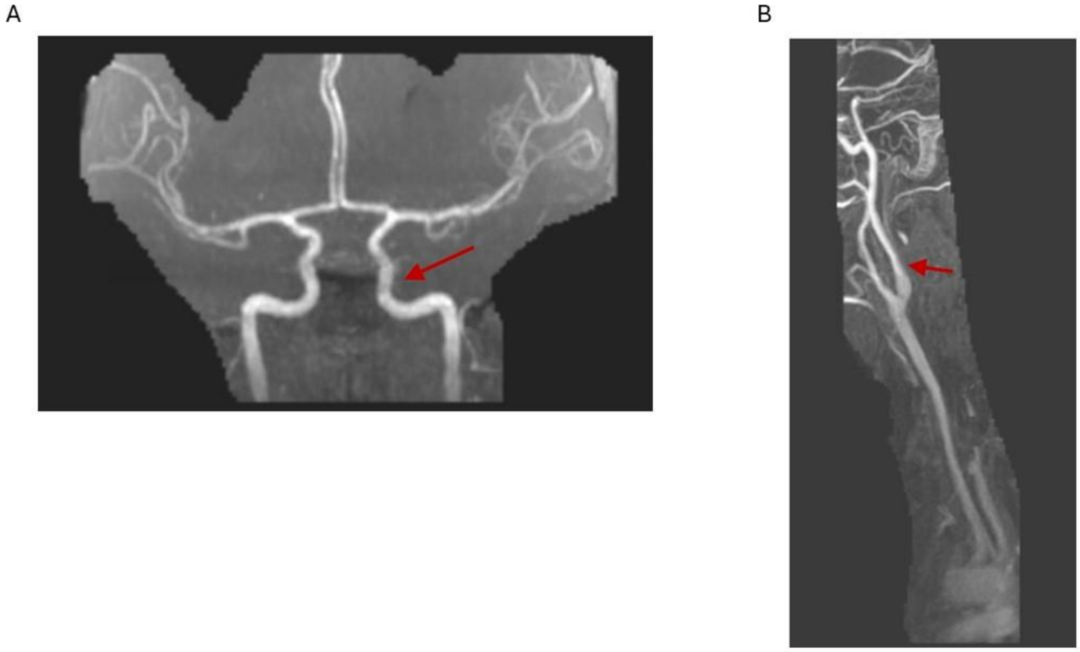

Supplementary Figure 1. Magnetic resonance angiogram of the head (panel A) and neck (panel B) without contrast in the coronal view at initial presentation. Red arrows indicate no evidence of dissection in the left mid-to-distal internal carotid artery.

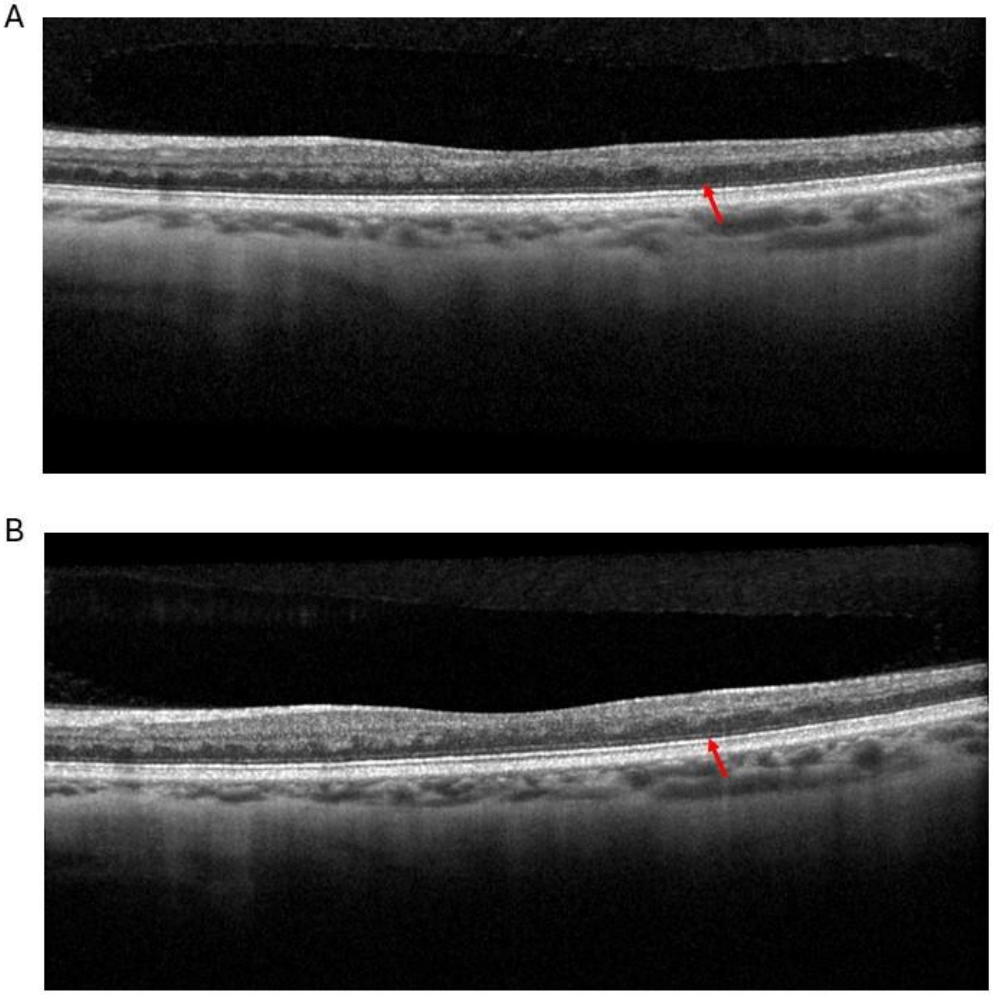

Supplementary Figure 2. Subsequent spectral-domain optical coherence tomography images following the acute ischemic period show resolution of the acute hyperreflective paracentral acute middle maculopathy lesion which has been supplanted by attenuation of the inner nuclear layer: 3 months after initial presentation (April 2024) (panel A) and 7 months after initial presentation (August 2024) (panel B).
